# Evaluation of Scientific Quality of YouTube Video Content Related to Umbilical Hernia

**DOI:** 10.7759/cureus.14675

**Published:** 2021-04-25

**Authors:** Guner Cakmak

**Affiliations:** 1 General Surgery, Sakarya Training and Research Hospital, Sakarya, TUR

**Keywords:** umbilical hernia, internet, youtube, quality, discern, gqs

## Abstract

Objective: Patients with umbilical hernias frequently refer to the YouTube videos to learn and perhaps apply traditional treatment methods. It is very difficult for these users to distinguish these videos as useful or harmful. In this study, we aimed to evaluate the scientific quality of YouTube video content on umbilical hernia.

Methods: A total of 50 videos on YouTube pertaining to umbilical hernia were included in the study. All videos were evaluated by two experienced general surgeons. The uploader, video content, length, upload date, time since upload, number of views, numbers of comments, likes, and dislikes and Video Power Index (VPI) rates videos were recorded and evaluated. The videos were scored using the Quality Criteria for Consumer Health Information (DISCERN) and Global Quality Scale (GQS).

Results: A total of 9,836 comments were made to the videos, 118,478 likes were made, and 15,009 dislikes were made. The mean DISCERN score given to the videos by the researchers was 2.57 ± 1.82 (min-max: 1-5) and the average GQS score was 2.62 ± 1.86. A statistically significant difference was found in terms of both DISCERN and GQS scores of videos uploaded by doctors compared to videos uploaded by nondoctors (p < 0.001). A statistically significant level of good agreement was found among investigators in terms of both DISCERN (p < 0.001, r = 0.778) and GQS (p < 0.001, r = 0.807) scores.

Conclusion: Videos with health content should definitely be uploaded by experts. Studies investigating the scientific quality of health videos uploaded on YouTube and similar platforms should be carried out continuously.

## Introduction

An umbilical hernia is a condition in which the abdominal wall behind the navel is damaged. Umbilical hernia is manifested by protrusion or swelling from the umbilicus. The occurrence of umbilical hernia may be due to some congenital anatomical features, as well as from heavy lifting, carrying loads, prolonged physical activity, and sudden movements. Umbilical hernia is the second most common hernia type after inguinal hernia in adults [1], and it accounts for 6%-14% of all abdominal wall hernias [2].

Umbilical hernia is popularly known as belly drop, navel shift, or abduction of belly. This disease is tried to be treated traditionally with methods such as cupping with blood-letting, only cupping method, belly massage, tying rope, stone or money to the navel [3]. Although these methods can provide a short term relief of the complaints, when the umbilical hernia is not treated, it causes a swelling consisting of abdominal fat, omentum and partially small intestines in the navel, pain and especially some serious intestinal diseases [4]. Patients go to hospitals with complaints of abdominal pain, nausea, and vomiting. Definitive treatment of umbilical hernia is surgical. However, patients with umbilical hernias frequently refer to the Internet to learn and perhaps apply the traditional "navel drop" treatment methods. Fear of surgery, also leads these patients to seek alternative and noninvasive treatment methods.

The Internet is a frequently used resource by individuals all around the world to search for information about health issues [5]. The most widely used Internet platform is Google (www.google.com) and the most widely used video sharing platform is YouTube (www.youtube.com). Regarding educational and useful information about health on YouTube, the number of videos containing misleading information is quite high [6-9]. It is very difficult for users to distinguish these videos as useful or harmful. The number of health content uploaded on YouTube is rapidly increasing [10], but there is no preliminary assessment in terms of the accuracy and reliability of this content, and anyone can upload such content freely and free of charge. This situation has led health professionals to conduct studies investigating the quality and reliability of these uploaded contents [11-12]. There have been many studies investigating the quality of health videos found in almost every field of medicine on YouTube [6-9, 11, 13]. However, in a literature search we did not find a study investigating the videos containing umbilical hernia.

In this study, we aimed to evaluate the scientific quality of YouTube video content on umbilical hernia.

## Materials and methods

Study design and collection of data

The study was designed as an observational study by two general surgeons. The first researcher is a specialist physician with 22 years of general surgery experience. The second researcher is a specialist physician with 12 years of general surgery experience. The keywords "umbilical hernia", "navel displacement," and "navel shift" were used in our study. Key terms were determined using Google Trends (www.trends.google.com) application [14]. On 15.03.2021, the keywords were entered separately in the YouTube search bar and the "relevance" was selected by using the filtering feature. A total of 110 videos those are most relevant for three search terms are listed. Among these videos, after excluding those shorter than 60 s, those with advertisements, those longer than 20 min, those with entertainment purposes, repetitive videos, and non-English videos, the remaining 50 videos were included in the study.

The uploader, video content, length (minutes), upload date, time since upload (days), number of views, number of comments, number of likes, number of dislikes, and Video Power Index (VPI) rates of the 50 videos that meet the inclusion criteria have been recorded to an Excel file. The quality of the videos was evaluated by VPI values calculated according to the formula: VPI = like count / (like count + dislike count) x 100 [8-9]. Later, these videos were evaluated by two researchers in separate environments.

Evaluation of the videos

**Figure 1:**
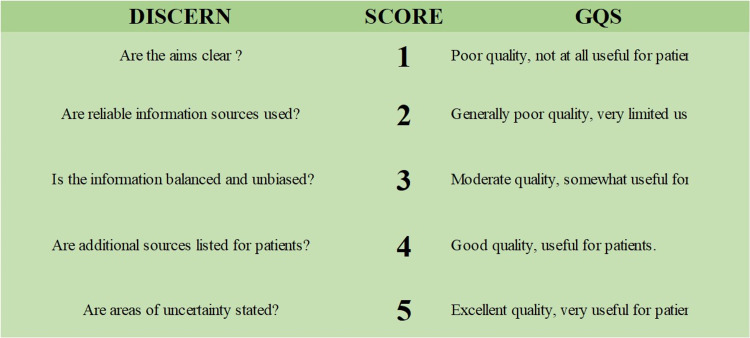
Questions concerning DISCERN and GQS scales. DISCERN: Quality Criteria for Consumer Health Information [16], GQS: Global Quality Scale [17].

The videos included in the study were scored according to the Quality Criteria for Consumer Health Information (DISCERN) and Global Quality Scale (GQS), which were used in many studies before. The DISCERN scale was developed by Charnock et al. [15]. The DISCERN scale, configured by Singh et al., is used to evaluate the reliability of videos [16]. There are a total of five questions in the scale, and high scores show that the videos have reliable content. The GQS scale was used to determine the quality of the videos [17]. The scale consists of five questions -- one point indicates very poor quality, two points for poor quality and limited use, three points for medium quality, four points for good quality, and five points for very good quality. Questions of DISCERN and GQS scales are given in Figure 1.

In our study, we evaluated one to two points from both DISCERN and GQS scales as bad quality and misleading content, three points as medium quality content, and four to five points as useful and high quality content.

Statistical analysis

Data obtained in this study were statistically analyzed using SPSS version 23.0 (SPSS, Statistical Package for Social Sciences, IBM Inc., Chicago, IL, USA). Normality of the data was evaluated with the Kolmogorov-Smirnov test. The variables were expressed as mean ± standard deviation, numbers, and percentage. The comparison between the videos uploaded by doctors and nondoctors was made utilizing the Mann-Whitney U test. The agreement between the two observers was analyzed with the Spearman’s correlation analysis. p < 0.05 values were considered statistically significant.

## Results

A total of 110 videos were examined in our study. After excluding repeated videos, commercial videos and off-topic videos, the remaining 50 videos were included in the study. It was determined that the total number of views of all videos was 24,846,705. The general characteristics of the videos according to the nature of the uploaders and the video content are given in Table 1.

**Table 1:** The nature of the uploaders of the videos and the distribution of the video content.

		Video content						
Uploader	n	Surgical technique	General information	Neural therapy	Yoga	Exercise	Cupping therapy	Other
Doctor	10	5	5	0	0	0	0	0
Hospital channel	4	1	3	0	0	0	0	0
Health channel	7	4	3	0	0	0	0	0
Neural therapist	12	0	0	10	0	0	1	1
Yoga instructor	8	0	0	0	7	0	0	1
Masseur	4	0	0	0	0	1	2	1
Other	5	0	0	0	0	3	1	1
Total	50	10	11	10	7	4	4	4

When Table 1 was examined, it was observed that all surgical technique and general information videos were uploaded by doctors, hospital channels and health channels, while methods such as neurofeedback, yoga, exercise, and cupping therapy were loaded by other users. The number of comments, likes, and dislikes of all videos according to the nature of the uploaders are given in Table 2.

**Table 2:** Number of comments, likes, and dislikes according to uploaders of videos.

Video uploader	Viewing	n	Comments	Like	Dislike
Doctor	2,064,172	10	466	13,694	1,003
Hospital channel	1,220,292	4	235	8,995	688
Health channel	5,739,789	7	277	4,261	439
Neural therapist	12,602,282	12	4,974	77,037	10,685
Yoga instructor	1,169,575	8	1,729	2,539	813
Masseur	251,013	4	892	2,273	248
Other	1,799,582	5	1,263	9,679	1,133
Total	24,846,705	50	9,836	118,478	15,009

When Table 2 is examined, it is seen that a total of 9,836 comments were made to the videos, 118,478 likes were made, and 15,009 dislikes were made. It was determined that the most viewed video was uploaded by a neural therapist in 2018 and watched a total of 7,949,777 times, received 826 comments, 24,000 likes, and 7,100 dislikes.

The average number of views of the 50 videos examined in the study, the time elapsed after the date the videos were uploaded, the average of daily views, comments, like, dislike and VPI are given in Table 3.

**Table 3:** General features and the features according to doctor and nondoctor uploaders of the videos. VPI, Video Power Index; SD, standard deviation

	All videos	Doctor	Nondoctor
	Mean ± SD	Mean ± SD	Mean ± SD
Video length (min)	7.56 ± 4.40	6.30 ± 4.20	6.53 ± 4.22
Viewing count	496,934 ± 1,272,483	206,417 ± 1,272,483	569,563 ± 1,308,860
Time elapsed	1147.5 ± 761.41	1474.8 ± 761.4	1.065 ± 755
Daily viewing	58,819 ± 1,433	23,583 ± 1,433,86	676 ± 1,472
Comments	197 ± 300	47 ± 300	235 ± 308
Likes	2,632 ± 4,469	1,369 ± 4,568	2,948 ± 4,680
Dislikes	300 ± 1,005	1,003 ± 1,005	350 ± 1,035
VPI (%)	90.25 ± 8.0	89.33 ± 8.32	90.48 ± 8.46

The average DISCERN score given to the videos by the researchers was 2.57 ± 1.82 (min-max: 1-5) and the average GQS score was 2.62 ± 1.86. The average DISCERN score of the first researcher was 2.67 ± 1.86 and the average GQS score was 2.66 ± 1.91. Similarly, the mean DISCERN score of the second investigator was 2.54 ± 1.78 and the average GQS score was 2.58 ± 1.82. Average DISCERN and GQS scores of the researchers according to the quality of the uploaders of the videos are given in Table 4.

**Table 4:** DISCERN and GQS scores according to the nature of the video uploaders.

	n	DISCERN 1	GQS 1	DISCERN 2	GQS 2
Doctor	10	4.9 ± 1.86	5.0 ± 1.91	4.7 ± 1.78	4.8 ± 1.82
Hospital channel	4	4.5 ± 0.77	4.5 ± 0.66	4 ± 0.60	4.2 ± 0.52
Health channel	7	4 ± 1.81	4.1 ± 1.86	4.1 ± 1.78	4.1 ± 1.86
Neural therapist	12	1.33 ± 0.74	1.33 ± 0.74	1.25 ± 0.55	1.25 ± 0.55
Yoga instructor	8	1 ± 0	1 ± 0	1 ± 0	1 ± 0
Masseur	4	1 ± 0	1 ± 0	1 ± 0	1 ± 0
Other	5	1 ± 0	1 ± 0	1 ± 0	1 ± 0

Examining Table 4, 21 videos (42%) uploaded by doctors (20%), hospital channels (8%), and health channels (14%) were found to be of excellent quality and useful videos. However, 29 videos (58%) uploaded by other users were found to have very poor quality and misleading content.

When we divided the videos into two groups as doctors (20%) and nondoctors (80%) according to the quality of the uploaders, the average DISCERN score of the videos uploaded by the doctors was 4.8 ± 1.88 and the average GQS score was 4.97 ± 1.92. The average DISCERN score of videos uploaded by nondoctors was 2.1 ± 1.73 and the average GQS score was 2.1 ± 1.78. Accordingly, a statistically significant difference was found in terms of both DISCERN and GQS scores of videos uploaded by doctors compared to videos uploaded by nondoctors (p < 0.001). A statistically significant level of good agreement was found among investigators in terms of both DISCERN (p < 0.001, r = 0.778) and GQS (p < 0.001, r = 0.807) scores.

## Discussion

YouTube is the largest video sharing site with two billion active users and providing free access to its users all over the world [18]. A study reported that eight out of every 10 people search for information about their health on the Internet [19]. This shows the importance of health information on large platforms such as YouTube. Many studies have been conducted previously to evaluate the videos on YouTube [6-9, 16]. However, we did not find a study evaluating umbilical hernia videos. For this reason, we aimed to evaluate the quality of umbilical hernia videos on YouTube.

We found that 50 videos we analyzed were viewed 24,846,705 times. Only 10 (20%) of these videos were uploaded by doctors and viewed 2,064,172 times in total. The videos uploaded by the doctors mainly contained general information and surgical technique about umbilical hernia. Twelve videos were uploaded and viewed 12,602,282 times by neural therapists. Ten of these 12 videos were about neural therapy, one video for cupping treatment, and one video was about a different method. This result shows us that videos uploaded by nonphysicians are viewed more than videos uploaded by doctors. In similar studies examining YouTube videos, it was reported that videos uploaded by nonphysicians were viewed more [7-8, 20-21].

According to the scores given by the two researchers to the videos with the DISCERN and GQS scale, 21 (42%) of the 50 videos were found to have useful / quality content, and 29 (48%) were found to contain very bad / misleading information. YouTube videos with "inguinal hernia repair" content were evaluated by Keskinkılıç Yağız et al., and 13 videos (23.6%) were reported to be of good quality, 22 videos (40%) were of medium quality, and 20 videos (36.4%) were of poor quality [22]. Similarly, Reitano et al. evaluated the "trans abdominal pre-peritoneal hernia repair" videos on YouTube, and reported that 13 (65%) of the videos were bad, six (30%) were good, and one video (5%) was of high quality [23]. In our study, all useful videos were uploaded by doctors, hospital channels and health channels, while all misleading videos were uploaded by other users. Considering that the treatment method of umbilical hernia is a surgical procedure only, this result is not surprising. Because, in the videos uploaded by other users, umbilical hernia was named as navel drop or belly shift and primitive treatment methods were applied. In previous similar studies, it has also been reported that videos uploaded by nonphysicians are of poor quality and misleading [6-7, 20-21].

When the VPI values of the videos we examined were examined, the average VPI score of the videos uploaded by the doctors was 89.33 ± 8.32, while the average VPI score for the nonphysicians was found to be 90.48 ± 8.46. However, it was also found that the videos uploaded by nonphysicians were viewed more, liked more, and made more comments. In many studies analyzing YouTube videos, it has also been reported that the videos uploaded by nondoctors are of poor quality and misleading, but are more liked by users [24-25]. We think that the reason for this may be that the scientific expression and the language used in the videos uploaded by doctors is not understood by everyone and that patients and their relatives seek traditional treatment methods as an alternative to surgical intervention, which is the principal treatment of umbilical hernia.

It was determined that the most viewed one of the videos in our study, was uploaded by a neural therapist in 2018, viewed 7,949,777 times, and received 24,000 likes. The same video was given a score of one by the researchers on both DISCERN and GQS scales. The least watched (1,825 views) video was uploaded in 2020; it is an exercise content video and has been found to have poor quality and misleading content.

In this study, where we evaluated the quality and reliability of videos with umbilical hernia content, we found that videos uploaded by doctors, hospital channels, and health channels are useful videos. In similar studies in the literature, there are studies reporting that some of the videos uploaded by even the doctors are not of sufficient quality [7, 21]. In these studies, it is emphasized that the videos uploaded by doctors, hospital channels, or health channels should also pass through a control mechanism [20, 26]. We as well, think that health videos should only be uploaded by experts.

Limitations of the study

The main limitation of our study is that the data used in the study are obtained in a specific and limited time period. As is known, data about YouTube videos can change instantly. As another restriction, more videos could be included in the study. However, since the most watched videos are included in our study, it is clear that including more videos will have a disincentive potential. Nevertheless, strength of our study is that it is the first YouTube evaluation study on umbilical hernia, one of the most common health problems. We believe our study will encourage future studies on the quality of YouTube video content.

## Conclusions

In our study, we found that 29 of 50 videos with umbilical hernia content had very poor quality and misleading content. All videos with poor quality and misleading content were uploaded by irrelevant users. It has been determined that the videos uploaded by doctors, hospital channels, and health channels are of good quality and contain useful content. We think that videos with health content should definitely be uploaded by experts. In addition, we believe that studies investigating the scientific quality of health videos uploaded on YouTube and similar platforms should be carried out continuously.
